# Supplementary material for: Predicting drug–gene relations via analogy tasks with word embeddings
Source: Sci Rep. 2025 May 18;15:17240. doi: 10.1038/s41598-025-01418-z (PMC12086191; doi:10.1038/s41598-025-01418-z)
Supplement: Supplementary file 1 — Supplementary Information. [file 41598_2025_1418_MOESM1_ESM.pdf]

Predicting Drug-Gene Relations via Analogy Tasks with Word Embeddings

Hiroaki Yamagiwa[1,α,*], Ryoma Hashimoto[2,β], Kiwamu Arakane[3,γ], Ken Murakami[4,δ], Shou Soeda[3,ε], Momose Oyama[1,5,ζ], Yihua Zhu[1,η], Mariko Okada[3,θ], and Hidetoshi Shimodaira[1,5,ι]

[1]Kyoto University, Kyoto, Japan
[2]Recruit Co., Ltd., Tokyo, Japan
[3]Institute for Protein Research, Osaka University, Osaka, Japan
[4]Present address: Research Institute of Molecular Pathology, Vienna BioCenter, Vienna, Austria
[5]RIKEN, Tokyo, Japan
[α]h.yamagiwa@i.kyoto-u.ac.jp
[β]ryoma_hashimoto@r.recruit.co.jp
[γ]k.arakane@protein.osaka-u.ac.jp
[δ]ken.murakami@imp.ac.at
[ε]shousoeda@protein.osaka-u.ac.jp
[ζ]oyama.momose@sys.i.kyoto-u.ac.jp
[η]zhu.yihua.22h@st.kyoto-u.ac.jp
[θ]mokada@protein.osaka-u.ac.jp
[ι]shimo@i.kyoto-u.ac.jp
[*]Corresponding author

Supplementary Information

1 Details of analogy tasks

1.1 Analogy tasks for drug-gene pairs.

1.1.1 *Pathway-wise setting*

For easy comparison with Eq. (5), Eq. (14) is rewritten as the difference of mean vectors:

$$\hat{\mathbf{v}}_p = \mathrm{E}_{\mathscr{R}_p}\{\mathbf{u}_g\} - \mathrm{E}_{\mathscr{R}_p}\{\mathbf{u}_d\}, \tag{S1}$$

$$\mathrm{E}_{\mathscr{R}_p}\{\mathbf{u}_d\} = \frac{1}{|\mathscr{R}_p|}\sum_{(d,g)\in\mathscr{R}_p}\mathbf{u}_d, \quad \mathrm{E}_{\mathscr{R}_p}\{\mathbf{u}_g\} = \frac{1}{|\mathscr{R}_p|}\sum_{(d,g)\in\mathscr{R}_p}\mathbf{u}_g. \tag{S2}$$

In Eq. (14), the estimator $\hat{\mathbf{v}}_p$ for the relation vector $\mathbf{v}_p$ is calculated from the embeddings of the drug-gene pairs $(d,g) \in \mathscr{R}_p$, while, similar to Eq. (5), the estimator for $\mathbf{v}_p$ can also be calculated from the embeddings of $d \in \mathscr{D}_p$ and $g \in \mathscr{G}_p$. The following equation gives a naive estimator for $\mathbf{v}_p$:

$$\hat{\mathbf{v}}_{p,\mathrm{naive}} := \mathrm{E}_{\mathscr{G}_p}\{\mathbf{u}_g\} - \mathrm{E}_{\mathscr{D}_p}\{\mathbf{u}_d\}, \tag{S3}$$

$$\mathrm{E}_{\mathscr{D}_p}\{\mathbf{u}_d\} = \frac{1}{|\mathscr{D}_p|}\sum_{d\in\mathscr{D}_p}\mathbf{u}_d, \quad \mathrm{E}_{\mathscr{G}_p}\{\mathbf{u}_g\} = \frac{1}{|\mathscr{G}_p|}\sum_{g\in\mathscr{G}_p}\mathbf{u}_g. \tag{S4}$$

In Eq. (S4), $\mathrm{E}_{\mathscr{D}_p}\{\cdot\}$ and $\mathrm{E}_{\mathscr{G}_p}\{\cdot\}$ are the means over the set of all drugs $\mathscr{D}$ and the set of all genes $\mathscr{G}$, respectively. Equation (S3) represents the vector difference between the mean vectors of $\mathscr{D}_p$ and $\mathscr{G}_p$. However, similar to $\hat{\mathbf{v}}_{\mathrm{naive}}$ in Eq. (5), the definition of $\hat{\mathbf{v}}_{p,\mathrm{naive}}$ in Eq. (S3) includes the embeddings of unrelated genes and drugs. Therefore, in Supplementary Information 2.1.1, we compare $\hat{\mathbf{v}}$ and $\hat{\mathbf{v}}_{\mathrm{naive}}$, as well as $\hat{\mathbf{v}}_p$ and $\hat{\mathbf{v}}_{p,\mathrm{naive}}$.

Note the following in setting P2:

- Since $D_p \subset \mathscr{D}_p$ and $D_p = \pi_{\mathscr{D}_p}(\mathscr{R}_p) = \pi_{\mathscr{D}}(\mathscr{R}_p) \subset \pi_{\mathscr{D}}(\mathscr{R}) = D$, it follows that $D_p \subset \mathscr{D}_p \cap D$. For $d \in \mathscr{D}_p \cap D$, there may be $g \in [d] \setminus [d]_p$. Therefore, in Eq. (13), we consider $d \in \mathscr{D}_p \cap D$ and $g \in [d]$, instead of $(d,g) \in \mathscr{R}_p$.

Fig. S1 shows a specific example to illustrate the differences between settings P1 and P2. For drug-gene pairs with drug-gene relations, setting P1 focuses on drugs and genes categorized in the same pathway, while setting P2 considers those genes as well as genes not categorized in the pathway.

1.2 Analogy tasks for drug-gene pairs by year

1.2.1 Global setting by year

Similar to the estimator $\hat{\mathbf{v}}$ in Eq. (7), we define an estimator $\hat{\mathbf{v}}^y$ for the relation vector $\mathbf{v}^y$ as the mean of the vector differences $\mathbf{u}_g - \mathbf{u}_d$ for $(d,g) \in \mathscr{R}^y$:

$$\hat{\mathbf{v}}^y := \mathrm{E}_{\mathscr{R}^y}\{\mathbf{u}_g - \mathbf{u}_d\} = \frac{1}{|\mathscr{R}^y|} \sum_{(d,g)\in\mathscr{R}^y} (\mathbf{u}_g - \mathbf{u}_d), \tag{S5}$$

where $\mathrm{E}_{\mathscr{R}^y}\{\cdot\}$ is the sample mean over the set of drug-gene pairs $\mathscr{R}^y$. For easy comparison with Eq. (5), Eq. (S5) is rewritten as the difference of mean vectors:

$$\hat{\mathbf{v}}^y = \mathrm{E}_{\mathscr{R}^y}\{\mathbf{u}_g\} - \mathrm{E}_{\mathscr{R}^y}\{\mathbf{u}_d\}, \tag{S6}$$

$$\mathrm{E}_{\mathscr{R}^y}\{\mathbf{u}_d\} = \frac{1}{|\mathscr{R}^y|} \sum_{(d,g)\in\mathscr{R}^y} \mathbf{u}_d, \quad \mathrm{E}_{\mathscr{R}^y}\{\mathbf{u}_g\} = \frac{1}{|\mathscr{R}^y|} \sum_{(d,g)\in\mathscr{R}^y} \mathbf{u}_g. \tag{S7}$$

Similar to $\hat{\mathbf{v}}$ in Eq. (7), to measure the performance of the estimator $\hat{\mathbf{v}}^y$ in Eq. (S5), we prepare the evaluation of the analogy tasks. Using the projection operations in Eq. (10), we define $D^y := \pi_{\mathscr{D}^y}(\mathscr{R}^y) \subset \mathscr{D}^y$ and $G^y := \pi_{\mathscr{G}^y}(\mathscr{R}^y) \subset \mathscr{G}^y$.

Similar to Eq. (11), we define $[d]^y \subset \mathscr{G}^y$ as the set of genes that have drug-gene relations with a drug $d \in D^y$, and $[g]^y \subset \mathscr{D}^y$ as the set of drugs that have drug-gene relations with a gene $g \in G^y$. These are formally defined as follows:

$$[d]^y := \{g \mid (d,g) \in \mathscr{R}^y\} \subset \mathscr{G}^y, \quad [g]^y := \{d \mid (d,g) \in \mathscr{R}^y\} \subset \mathscr{D}^y. \tag{S8}$$

Given the above, we perform the analogy tasks in the following setting.

Setting Y1. In the analogy tasks, the set of answer genes for a query drug $d \in D^y$ is $[d]^y$. The predicted gene is $\hat{g}_d = \mathrm{argmax}_{g\in\mathscr{G}^y} \cos(\mathbf{u}_d + \hat{\mathbf{v}}^y, \mathbf{u}_g)$ and if $\hat{g}_d \in [d]^y$, then the prediction is considered correct. We define $\hat{g}_d^{(k)}$ as the k-th ranked $g \in \mathscr{G}^y$ based on $\cos(\mathbf{u}_d + \hat{\mathbf{v}}^y, \mathbf{u}_g)$. For the top-$k$ accuracy, if any of the top k predictions $\hat{g}_d^{(1)}, \dots, \hat{g}_d^{(k)} \in [d]^y$, then the prediction is considered correct.

1.2.2 Global setting to predict unknown relations by year

Similar to the estimator $\hat{\mathbf{v}}$ in Eq. (7), we define an estimator $\hat{\mathbf{v}}^{y|L_y}$ for the relation vector $\mathbf{v}^{y|L_y}$ as the mean of the vector differences $\mathbf{u}_g - \mathbf{u}_d$ for $(d,g) \in \mathscr{R}^{y|L_y}$:

$$\hat{\mathbf{v}}^{y|L_y} := \mathrm{E}_{\mathscr{R}^{y|L_y}}\{\mathbf{u}_g - \mathbf{u}_d\} = \frac{1}{|\mathscr{R}^{y|L_y}|} \sum_{(d,g)\in\mathscr{R}^{y|L_y}} (\mathbf{u}_g - \mathbf{u}_d), \tag{S9}$$

where $\mathrm{E}_{\mathscr{R}^{y|L_y}}\{\cdot\}$ is the sample mean over the set of drug-gene pairs $\mathscr{R}^{y|L_y}$. We define the estimator $\hat{\mathbf{v}}^{y|L_y}$ in Eq. (S9) by using $\mathscr{R}^{y|L_y}$ instead of $\mathscr{R}^y$ in the estimator $\hat{\mathbf{v}}^y$ in Eq. (S5). For easy comparison with Eq. (5), Eq. (S9) is rewritten as the difference of mean vectors:

$$\hat{\mathbf{v}}^{y|L_y} = \mathrm{E}_{\mathscr{R}^{y|L_y}}\{\mathbf{u}_g\} - \mathrm{E}_{\mathscr{R}^{y|L_y}}\{\mathbf{u}_d\}, \tag{S10}$$

$$\mathrm{E}_{\mathscr{R}^{y|L_y}}\{\mathbf{u}_d\} = \frac{1}{|\mathscr{R}^{y|L_y}|} \sum_{(d,g)\in\mathscr{R}^{y|L_y}} \mathbf{u}_d, \quad \mathrm{E}_{\mathscr{R}^{y|L_y}}\{\mathbf{u}_g\} = \frac{1}{|\mathscr{R}^{y|L_y}|} \sum_{(d,g)\in\mathscr{R}^{y|L_y}} \mathbf{u}_g. \tag{S11}$$

Similar to $\hat{\mathbf{v}}$ in Eq. (7), to measure the performance of the estimator $\hat{\mathbf{v}}^{y|L_y}$ in Eq. (S9), we prepare the evaluation of the analogy tasks. For $I \in \{L_y, U_y\}$, using the projection operations in Eq. (10), we define $D^{y|I} := \pi_{\mathscr{D}^y}(\mathscr{R}^{y|I}) \subset \mathscr{D}^y$ and $G^{y|I} := \pi_{\mathscr{G}^y}(\mathscr{R}^{y|I}) \subset \mathscr{G}^y$.

Similar to Eq. (11), for $I \in \{L_y, U_y\}$, we define $[d]^{y|I} \subset \mathscr{G}^y$ as the set of genes that have drug-gene relations with a drug $d \in D^{y|I}$, and $[g]^{y|I} \subset \mathscr{D}^y$ as the set of drugs that have drug-gene relations with a gene $g \in G^{y|I}$. These are formally defined as follows:

$$[d]^{y|I} := \{g \mid (d,g) \in \mathscr{R}^{y|I}\} \subset \mathscr{G}^y, \quad [g]^{y|I} := \{d \mid (d,g) \in \mathscr{R}^{y|I}\} \subset \mathscr{D}^y. \tag{S12}$$

Given the above, we perform the analogy tasks in the following setting.

	BioConceptVec	Our embeddings
$\|\mathscr{P}\|$	136	129
$\sum_{p\in\mathscr{P}} \|D_p\|$	4087	3612
$\sum_{p\in\mathscr{P}} \|G_p\|$	1251	1178
$\sum_{p\in\mathscr{P}} \|\mathscr{D}_p \cap D\|$	4091	3614
$\sum_{p\in\mathscr{P}} \|\mathscr{G}_p \cap G\|$	3463	3186
$\mathrm{E}_{p\in\mathscr{P}}\{\mathrm{E}_{d\in D_p}\{\|[d]_p\|\}\}$	1.965	1.990
$\mathrm{E}_{p\in\mathscr{P}}\{\mathrm{E}_{g\in G_p}\{\|[g]_p\|\}\}$	5.050	4.847
$\mathrm{E}_{p\in\mathscr{P}}\{\mathrm{E}_{d\in\mathscr{D}_p\cap D}\{\|[d]\|\}\}$	2.738	2.776
$\mathrm{E}_{p\in\mathscr{P}}\{\mathrm{E}_{g\in\mathscr{G}_p\cap G}\{\|[g]\|\}\}$	7.131	6.714

Table S1. Statistics for settings P1 and P2.

Setting Y2. For the target genes that have drug-gene relations with a drug d, only genes whose relations appeared after year y are considered correct. In other words, for a query drug $d \in D^{y|U_y}$, the set of answer genes is $[d]^{y|U_y}$. The search space is not the set of all genes $\mathscr{G}^y$, but the gene set $\mathscr{G}^y \setminus [d]^{y|L_y}$. The predicted gene is $\hat{g}_d = \mathrm{argmax}_{g\in\mathscr{G}^y\setminus[d]^{y|L_y}} \cos(\mathbf{u}_d + \hat{\mathbf{v}}^{y|L_y}, \mathbf{u}_g)$, and if $\hat{g}_d \in [d]^{y|U_y}$, then the prediction is considered correct. We define $\hat{g}_d^{(k)}$ as the k-th predicted gene, based on $\cos(\mathbf{u}_d + \hat{\mathbf{v}}^{y|L_y}, \mathbf{u}_g)$ for $g \in \mathscr{G}^y \setminus [d]^{y|L_y}$. For the top-$k$ accuracy, if $\hat{g}_d^{(k)} \in [d]^{y|U_y}$, then the prediction is considered correct.

Note the following in setting Y2:

- In setting Y2, the search space is $\mathscr{G}^y \setminus [d]^{y|L_y}$. This ensures that predicted target genes have the relations that appeared only after year y.

1.2.3 Pathway-wise setting by year

Based on the analogy tasks in settings P1, P2, Y1, and Y2, we consider the analogy tasks in the pathway-wise setting by year, where drugs and genes are categorized based on pathways in datasets divided by year.

Consider a fixed year y. When learning embeddings using PubMed abstracts up to year y as training data, we define $\mathscr{D}_p^y \subset \mathscr{D}^y$ and $\mathscr{G}_p^y \subset \mathscr{G}^y$ as the sets of drugs and genes that are categorized in each pathway $p \in \mathscr{P}$ and appeared up to year y, respectively. We then restrict the set $\mathscr{R}^y$ in Eq. (16) to each pathway p and define the subset of the set $\mathscr{R}_p^y$ as follows:

$$\mathscr{R}_p^y := \{(d,g) \in \mathscr{R}^y \mid d \in \mathscr{D}_p^y, g \in \mathscr{G}_p^y\} \subset \mathscr{D}_p^y \times \mathscr{G}_p^y. \tag{S13}$$

For drug-gene pairs $(d,g) \in \mathscr{R}_p^y$, we consider the analogy tasks for predicting the target genes g from a drug d. To solve these analogy tasks, we use the relation vector $\mathbf{v}_p^y$, which represents the relation between drugs and target genes categorized in the same pathway p. We predict $\mathbf{u}_g$ by adding the relation vector $\mathbf{v}_p^y$ to $\mathbf{u}_d$:

$$\mathbf{u}_d + \mathbf{v}_p^y \approx \mathbf{u}_g. \tag{S14}$$

Similar to equations (13) and (17), Eq. (S14) corresponds to Eq. (4). Therefore, an estimator $\hat{\mathbf{v}}_p^y$ for the relation vector $\mathbf{v}_p^y$ in Eq. (S14) is defined as follows:

$$\hat{\mathbf{v}}_p^y := \mathrm{E}_{\mathscr{R}_p^y}\{\mathbf{u}_g - \mathbf{u}_d\} = \frac{1}{|\mathscr{R}_p^y|} \sum_{(d,g)\in\mathscr{R}_p^y} (\mathbf{u}_g - \mathbf{u}_d). \tag{S15}$$

where $\mathrm{E}_{\mathscr{R}_p^y}\{\cdot\}$ is the sample mean over the set of drug-gene pairs $\mathscr{R}_p^y$. Similar to Eq. (7), Eq. (S15) defines the estimator $\hat{\mathbf{v}}_p^y$ as the mean of the vector differences $\mathbf{u}_g - \mathbf{u}_d$ for $(d,g) \in \mathscr{R}_p^y$. For easy comparison with Eq. (5), Eq. (S15) is rewritten as the difference of mean vectors:

$$\hat{\mathbf{v}}_p^y = \mathrm{E}_{\mathscr{R}_p^y}\{\mathbf{u}_g\} - \mathrm{E}_{\mathscr{R}_p^y}\{\mathbf{u}_d\}, \tag{S16}$$

$$\mathrm{E}_{\mathscr{R}_p^y}\{\mathbf{u}_d\} = \frac{1}{|\mathscr{R}_p^y|} \sum_{(d,g)\in\mathscr{R}_p^y} \mathbf{u}_d, \quad \mathrm{E}_{\mathscr{R}_p^y}\{\mathbf{u}_g\} = \frac{1}{|\mathscr{R}_p^y|} \sum_{(d,g)\in\mathscr{R}_p^y} \mathbf{u}_g. \tag{S17}$$

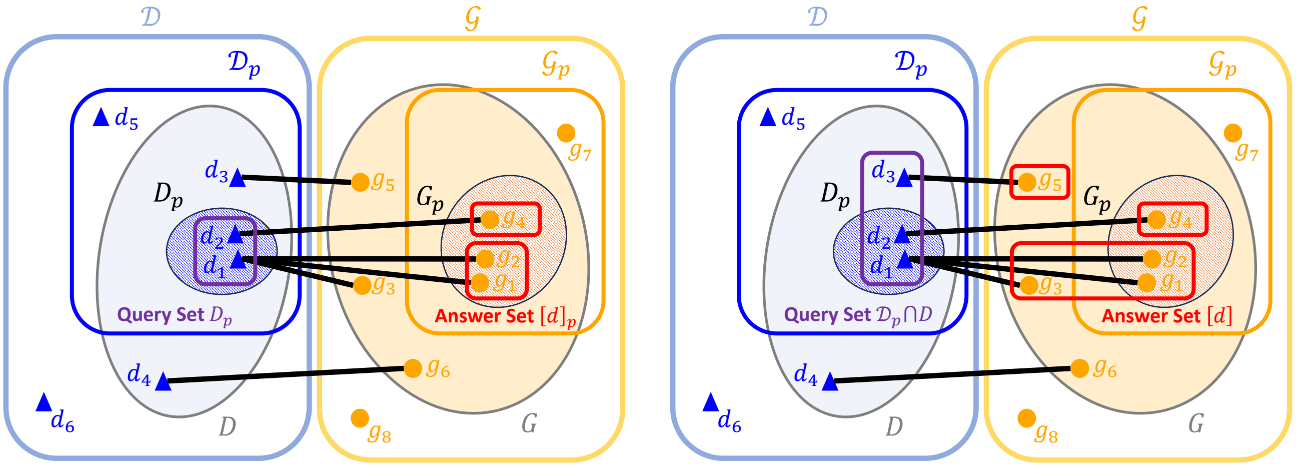

(a) P1 **(b)** P2

Figure S1. Differences between settings (a) P1 and (b) P2 using a specific example. We set $\mathscr{D} = \{d_1, d_2, d_3, d_4, d_5, d_6\}$, $\mathscr{G} = \{g_1, g_2, g_3, g_4, g_5, g_6, g_7, g_8\}$, and $\mathscr{R} = \{(d_1, g_1), (d_1, g_2), (d_1, g_3), (d_2, g_4), (d_3, g_5), (d_4, g_6)\}$. According to the definitions of D and G, $D = \{d_1, d_2, d_3, d_4\}$ and $G = \{g_1, g_2, g_3, g_4, g_5, g_6\}$. For the pathway p, we set $\mathscr{D}_p = \{d_1, d_2, d_3, d_5\}$ and $\mathscr{G}_p = \{g_1, g_2, g_4, g_7\}$. According to the definition of $\mathscr{R}_p$, $\mathscr{R}_p = \{(d_1, g_1), (d_1, g_2), (d_2, g_4)\}$. The definitions of D_p and G_p then give $D_p = \{d_1, d_2\}$ and $G_p = \{g_1, g_2, g_4\}$, based on $\mathscr{R}_p$. In setting P1, the query set is $D_p = \{d_1, d_2\}$, where the set of answer genes for d_1 is $[d_1]_p = \{g_1, g_2\}$ and for d_2 is $[d_2]_p = \{g_4\}$. In setting P2, the query set is $\mathscr{D}_p \cap D = \{d_1, d_2, d_3\}$, where the set of answer genes for d_1 is $[d_1] = \{g_1, g_2, g_3\}$, for d_2 is $[d_2] = \{g_4\}$, and for d_3 is $[d_3] = \{g_5\}$.

Similar to $\hat{\mathbf{v}}$ in Eq. (7), to measure the performance of the estimator $\hat{\mathbf{v}}_p^y$ in Eq. (S15), we prepare the evaluation of the analogy tasks. Using the projection operations in Eq. (10), we define $D_p^y := \pi_{\mathscr{D}_p^y}(\mathscr{R}_p^y)$ and $G_p^y := \pi_{\mathscr{G}_p^y}(\mathscr{R}_p^y)$.

Similar to Eq. (11), we define $[d]_p^y \subset \mathscr{G}_p^y$ as the set of genes that have drug-gene relations with a drug $d \in D_p^y$, and $[g]_p^y \subset \mathscr{D}_p^y$ as the set of drugs that have drug-gene relations with a gene $g \in G_p^y$. These are formally defined as follows:

$$[d]_p^y := \{g \mid (d, g) \in \mathscr{R}_p^y\} \subset \mathscr{G}_p^y, \quad [g]_p^y := \{d \mid (d, g) \in \mathscr{R}_p^y\} \subset \mathscr{D}_p^y. \tag{S18}$$

Similar to settings P1, P2, Y1, we perform the analogy tasks in the following four settings.

Setting P1Y1. For the target genes that have drug-gene relations with a drug d, only genes categorized in the same pathway p as the drug d are considered correct. In other words, for a query drug $d \in D_p^y$, the set of answer genes is $[d]_p^y$. The search space is the set of all genes $\mathscr{G}^y$, not limited to $\mathscr{G}_p^y$, the set of genes categorized in the pathway p. The predicted gene is $\hat{g}_d = \text{argmax}_{g \in \mathscr{G}^y} \cos(\mathbf{u}_d + \hat{\mathbf{v}}_p^y, \mathbf{u}_g)$, and if $\hat{g}_d \in [d]_p^y$, then the prediction is considered correct. We define $\hat{g}_d^{(k)}$ as the k-th ranked $g \in \mathscr{G}^y$ based on $\cos(\mathbf{u}_d + \hat{\mathbf{v}}_p^y, \mathbf{u}_g)$. For the top-$k$ accuracy, if $\hat{g}_d^{(k)} \in [d]_p^y$, then the prediction is considered correct.

Setting P2Y1. The gene predictions $\hat{g}_d$ and $\hat{g}_d^{(k)}$ are defined exactly the same as those in setting P1Y1, but the answer genes are defined the same as in setting Y1. That is, for the target genes that have drug-gene relations with a drug d, genes are considered correct regardless of whether they are categorized in the same pathway p as the drug d or not. In other words, for a query drug $d \in D^y$, the set of answer genes is $[d]^y$, and the prediction is considered correct if $\hat{g}_d \in [d]^y$. For the top-k accuracy, if $\hat{g}_d^{(k)} \in [d]^y$, then the prediction is considered correct. Note that the experiment is performed for $d \in \mathscr{D}_p^y \cap D^y$ for each p.

1.2.4 Pathway-wise setting to predict unknown relations by year

For $(d, g) \in \mathscr{R}_p^y$, we define two subsets of $\mathscr{R}_p^y$ based on whether $y_{(d,g)} \leq y$ or $y < y_{(d,g)}$. Similar to $\mathscr{R}^{y|L_y}$ and $\mathscr{R}^{y|U_y}$ in Eq. (18), using $L_y = (-\infty, y]$ and $U_y = (y, \infty)$, we define the set $\mathscr{R}_p^{y|L_y}$ and $\mathscr{R}_p^{y|U_y}$ as follows:

$$\mathscr{R}_p^{y|L_y} := \{(d, g) \in \mathscr{R}_p^y \mid y_{(d,g)} \in L_y\}, \mathscr{R}_p^{y|U_y} := \{(d, g) \in \mathscr{R}_p^y \mid y_{(d,g)} \in U_y\} \subset \mathscr{R}_p^y. \tag{S19}$$

The set of drug-gene pairs that satisfies $y_{(d,g)} \leq y$ is $\mathscr{R}_p^{y|L_y}$, and the set of drug-gene pairs that satisfies $y < y_{(d,g)}$ is $\mathscr{R}_p^{y|U_y}$. By definition, $\mathscr{R}_p^{y|L_y} \cap \mathscr{R}_p^{y|U_y} = \emptyset$ and $\mathscr{R}_p^{y|L_y} \cup \mathscr{R}_p^{y|U_y} \subset \mathscr{R}_p^y$.

In analogy tasks, we use "known" $\mathscr{R}_p^{y|L_y}$ and then predict the target genes g from a drug d for (d,g) in "unknown" $\mathscr{R}_p^{y|U_y}$. Using the vector $\mathbf{v}_p^{y|L_y}$, which represents the drug-gene relations and is derived from $\mathscr{R}_p^{y|L_y}$, we predict $\mathbf{u}_g$ by adding the relation vector $\mathbf{v}_p^{y|L_y}$ to $\mathbf{u}_d$:

$$\mathbf{u}_d + \mathbf{v}_p^{y|L_y} \approx \mathbf{u}_g. \tag{S20}$$

Similar to equations (13) and (19), Eq. (S20) corresponds to Eq. (4). Therefore, an estimator $\hat{\mathbf{v}}_p^{y|L_y}$ for the relation vector $\mathbf{v}_p^{y|L_y}$ in Eq. (S20) is defined as follows:

$$\hat{\mathbf{v}}_p^{y|L_y} := \mathrm{E}_{\mathscr{R}_p^{y|L_y}}\{\mathbf{u}_g - \mathbf{u}_d\} = \frac{1}{\left|\mathscr{R}_p^{y|L_y}\right|} \sum_{(d,g)\in\mathscr{R}_p^{y|L_y}} (\mathbf{u}_g - \mathbf{u}_d). \tag{S21}$$

where $\mathrm{E}_{\mathscr{R}_p^{y|L_y}}\{\cdot\}$ is the sample mean over the set of drug-gene pairs $\mathscr{R}_p^{y|L_y}$. Similar to Eq. (7), Eq. (S21) defines the estimator $\hat{\mathbf{v}}_p^{y|L_y}$ as the mean of the vector differences $\mathbf{u}_g - \mathbf{u}_d$ for $(d,g) \in \mathscr{R}_p^{y|L_y}$. For easy comparison with Eq. (5), Eq. (S21) is rewritten as the difference of mean vectors:

$$\hat{\mathbf{v}}_p^{y|L_y} = \mathrm{E}_{\mathscr{R}_p^{y|L_y}}\{\mathbf{u}_g\} - \mathrm{E}_{\mathscr{R}_p^{y|L_y}}\{\mathbf{u}_d\}, \tag{S22}$$

$$\mathrm{E}_{\mathscr{R}_p^{y|L_y}}\{\mathbf{u}_d\} = \frac{1}{|\mathscr{R}_p^{y|L_y}|} \sum_{(d,g)\in\mathscr{R}_p^{y|L_y}} \mathbf{u}_d, \quad \mathrm{E}_{\mathscr{R}_p^{y|L_y}}\{\mathbf{u}_g\} = \frac{1}{|\mathscr{R}_p^{y|L_y}|} \sum_{(d,g)\in\mathscr{R}_p^{y|L_y}} \mathbf{u}_g. \tag{S23}$$

Similar to $\hat{\mathbf{v}}$ in Eq. (7), to measure the performance of the estimator $\hat{\mathbf{v}}_p^{y|L_y}$ in Eq. (S21), we prepare the evaluation of the analogy tasks. For $I \in \{L_y, U_y\}$, using the projection operations in Eq. (10), we define $D_p^{y|I} := \pi_{\mathscr{D}_p^y}(\mathscr{R}_p^{y|I})$ and $G_p^{y|I} := \pi_{\mathscr{G}_p^y}(\mathscr{R}_p^{y|I})$.

Similar to Eq. (11), for $I \in \{L_y, U_y\}$, we define $[d]_p^{y|I} \subset \mathscr{G}_p^y$ as the set of genes that have drug-gene relations with a drug $d \in D_p^{y|I}$, and $[g]_p^{y|I} \subset \mathscr{D}_p^y$ as the set of drugs that have drug-gene relations with a gene $g \in G_p^{y|I}$. These are formally defined as follows:

$$[d]_p^{y|I} := \{g \mid (d,g) \in \mathscr{R}_p^{y|I}\} \subset \mathscr{G}_p^y, \quad [g]_p^{y|I} := \{d \mid (d,g) \in \mathscr{R}_p^{y|I}\} \subset \mathscr{D}_p^y. \tag{S24}$$

Similar to settings P1, P2, Y2, we perform the analogy tasks in the following four settings.

Setting P1Y2. For the target genes that have drug-gene relations with a drug d, only genes that are categorized in the same pathway p as d and whose relations appeared after year y are considered correct. In other words, for a query drug $d \in D_p^{y|U_y}$, the set of answer genes is $[d]_p^{y|U_y}$. The search space is not the set of all genes $\mathscr{G}^y$, but the gene set $\mathscr{G}^y \setminus [d]_p^{y|L_y}$. The predicted gene is $\hat{g}_d = \mathrm{argmax}_{g\in\mathscr{G}^y\setminus[d]_p^{y|L_y}} \cos(\mathbf{u}_d + \hat{\mathbf{v}}_p^{y|L_y}, \mathbf{u}_g)$, and if $\hat{g}_d \in [d]_p^{y|U_y}$, then the prediction is considered correct. We define $\hat{g}_d^{(k)}$ as the k-th predicted gene, based on $\cos(\mathbf{u}_d + \hat{\mathbf{v}}_p^{y|L_y}, \mathbf{u}_g)$ for $g \in \mathscr{G}^y \setminus [d]_p^{y|L_y}$. For the top-$k$ accuracy, if $\hat{g}_d^{(k)} \in [d]_p^{y|U_y}$, then the prediction is considered correct.

Setting P2Y2. For the target genes that have drug-gene relations with a drug d, only genes whose relations appeared after year y are considered correct, regardless of whether they are categorized in the same pathway p as the drug d or not. In other words, for a query drug $d \in \mathscr{D}_p^y \cap D^{y|U_y}$, the set of answer genes is $[d]^{y|U_y}$. The search space is not the set of all genes $\mathscr{G}^y$, but the gene set $\mathscr{G}^y \setminus [d]^{y|L_y}$. The predicted gene is $\hat{g}_d = \mathrm{argmax}_{g\in\mathscr{G}^y\setminus[d]^{y|L_y}} \cos(\mathbf{u}_d + \hat{\mathbf{v}}_p^{y|L_y}, \mathbf{u}_g)$, and if $\hat{g}_d \in [d]^{y|U_y}$, then the prediction is considered correct. We define $\hat{g}_d^{(k)}$ as the k-th predicted gene, based on $\cos(\mathbf{u}_d + \hat{\mathbf{v}}_p^{y|L_y}, \mathbf{u}_g)$ for $g \in \mathscr{G}^y \setminus [d]^{y|L_y}$. For the top-$k$ accuracy, if $\hat{g}_d^{(k)} \in [d]^{y|U_y}$, then the prediction is considered correct.

Note the following in settings P1Y2 and P2Y2:

- In setting P1Y2, the search space is $\mathscr{G}^y \setminus [d]_p^{y|L_y}$. This ensures that predicted target genes, which are categorized in the same pathway p as the drug d, have the relations that appeared only after year y.
- In setting P2Y2, since $D_p^{y|U_y} \subset \mathscr{D}_p^y$ and $D_p^{y|U_y} = \pi_{\mathscr{D}_p^y}\left(\mathscr{R}_p^{y|U_y}\right) = \pi_{\mathscr{D}^y}\left(\mathscr{R}_p^{y|U_y}\right) \subset \pi_{\mathscr{D}^y}\left(\mathscr{R}^{y|U_y}\right) = D^{y|U_y}$, it follows that $D_p^{y|U_y} \subset \mathscr{D}_p^y \cap D^{y|U_y}$. For $d \in \mathscr{D}_p^y \cap D^{y|U_y}$, there may be $g \in [d]^{y|U_y} \setminus [d]_p^{y|U_y}$. Therefore, in Eq. (S14), we consider $d \in \mathscr{D}_p^y \cap D^{y|U_y}$ and $g \in [d]^{y|U_y}$, instead of $(d,g) \in \mathscr{R}_p^{y|U_y}$. The search space is $\mathscr{G}^y \setminus [d]^{y|L_y}$. This ensure that predicted target genes have the relations that appeared only after year y.

Setting	Query	Answer Set	Search Space
G	$d \in D$	$[d]$	$\mathscr{G}$
P1	$d \in D_p$	$[d]_p$	$\mathscr{G}$
P2	$d \in \mathscr{D}_p \cap D$	$[d]$	$\mathscr{G}$
Y1	$d \in D^y$	$[d]^y$	$\mathscr{G}^y$
Y2	$d \in D^{y\|U_y}$	$[d]^{y\|U_y}$	$\mathscr{G}^y \setminus [d]^{y\|L_y}$
P1Y1	$d \in D_p^y$	$[d]_p^y$	$\mathscr{G}^y$
P2Y1	$d \in \mathscr{D}_p^y \cap D^y$	$[d]^y$	$\mathscr{G}^y$
P1Y2	$d \in D_p^{y\|U_y}$	$[d]_p^{y\|U_y}$	$\mathscr{G}^y \setminus [d]_p^{y\|L_y}$
P2Y2	$d \in \mathscr{D}_p^y \cap D^{y\|U_y}$	$[d]^{y\|U_y}$	$\mathscr{G}^y \setminus [d]^{y\|L_y}$

Table S2. Query, answer set, and search space for each setting for predicting genes from drugs.

Setting	Query	Answer Set	Search Space
G'	$g \in G$	$[g]$	$\mathscr{D}$
$\mathrm{P1}'$	$g \in G_p$	$[g]_p$	$\mathscr{D}$
$\mathrm{P2}'$	$g \in \mathscr{G}_p \cap G$	$[g]$	$\mathscr{D}$

Table S3. Query, answer set, and search space for each setting for predicting drugs from genes.

1.3 Comparison of experimental settings

Table S2 shows the settings for predicting genes from drugs, as used in the main text. Similarly, Table S3 shows the settings for predicting drugs from genes. In Table S3, we adapted the notations used for predicting genes from drugs in Table S2 to those used for predicting drugs from genes, denoting them as G', $\mathrm{P1}'$, and $\mathrm{P2}'$.

1.4 Embeddings

Figure S2 shows the distribution of the sizes of the answer sets for each drug d in settings G, P1, and P2 for BioConceptVec and our skip-gram embeddings. Similarly, Fig. S3 shows the distribution of the sizes of the answer sets for each gene g in settings G', $\mathrm{P1}'$, and $\mathrm{P2}'$ for BioConceptVec and our skip-gram embeddings. The hyperparameters used to train our skip-gram are shown in Table S4.

1.5 Datasets

In the BioConceptVec vocabulary, genes are represented by gene IDs[1] and drugs are represented by MeSH (Medical Subject Headings, `https://www.nlm.nih.gov/mesh/meshhome.html`) IDs. Therefore, a conversion from IDs to names is necessary when performing experiments. In addition, in the data obtained from AsuratDB and the KEGG API, genes are represented by gene IDs and drugs are represented by KEGG IDs. Therefore, a conversion from KEGG IDs to MeSH IDs is also required for use with BioConceptVec. The procedures for converting these IDs are described in the following sections.

1.5.1 Conversion from MeSH ID to drug name

The BioConceptVec vocabulary registers drugs using MeSH (Medical Subject Headings, `https://www.nlm.nih.gov/mesh/meshhome.html`) IDs. This is due to the normalization of drug names using MeSH IDs in PubTator. Therefore, we explain the procedure for converting MeSH IDs to drug names. We used MeSH SPARQL (`https://hhs.github.io/meshrdf/sparql-and-uri-requests`) to obtain the MeSH headings corresponding to the MeSH IDs in the BioConceptVec vocabulary. We used these headings to convert MeSH IDs to drug names. We also applied this conversion to drugs in the vocabulary of our trained skip-gram model.

1.5.2 Conversion from gene ID to gene name

The BioConceptVec vocabulary registers genes using gene IDs. Therefore, we explain the procedure for converting gene IDs to gene names. We used the KEGG API to obtain the names corresponding to the gene IDs in the BioConceptVec vocabulary. This API allows for batch requests; for example, to process gene IDs 20, 21, 22, 23, 24, the data is available at `https://rest.kegg.jp/list/hsa:20+hsa:21+hsa:22+hsa:23+hsa:24`. If multiple names corresponded to

Hyperparameter	Values
Training epochs	10
Down-sampling threshold	10^{-5}
Learning rate	0.025
Window size	5
Negative samples	5
Minimal word occurrence	30
Dimension	300

Table S4. Hyperparameter for our skip-gram.

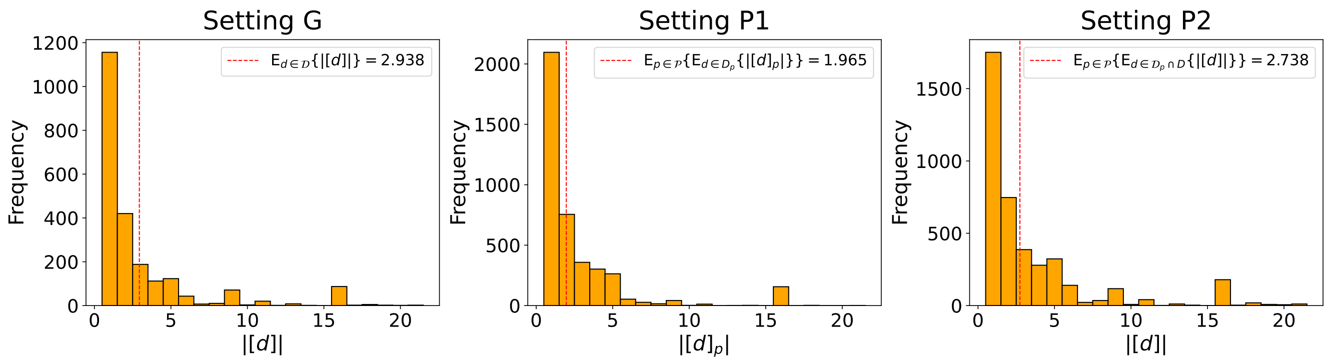

(a) BioConceptVec

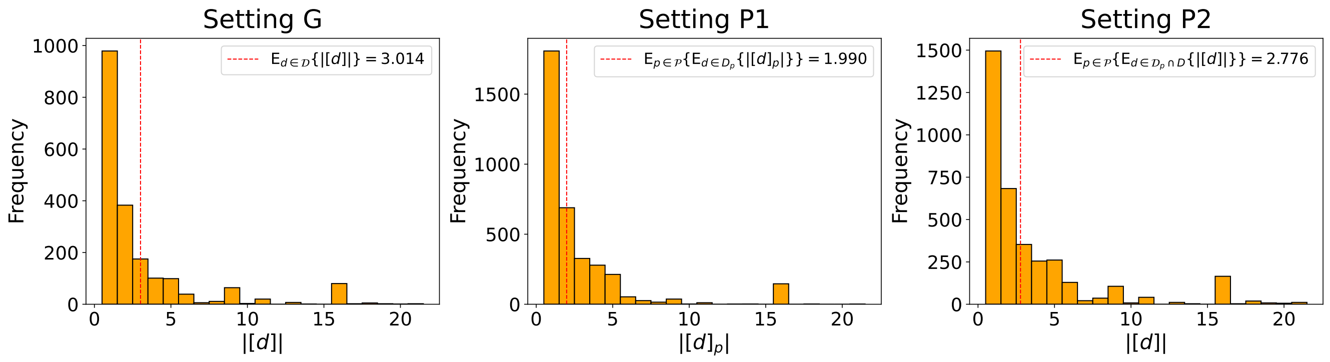

(b) Our embeddings

Figure S2. Distribution of the sizes of the answer sets for each drug d in the settings for predicting genes from drugs. The mean values are also shown: for setting G, the value of $\mathrm{E}_{d \in D}\{|[d]|\}$ from Table 2; for setting P1, the value of $\mathrm{E}_{p \in \mathscr{P}}\{\mathrm{E}_{d \in D_p}\{|[d]_p|\}\}$ from Table S1; and for setting P2, the value of $\mathrm{E}_{p \in \mathscr{P}}\{\mathrm{E}_{d \in \mathscr{D}_p \cap D}\{|[d]|\}\}$ from Table S1.

a single gene ID, we chose the first name. We also applied this conversion to genes in the vocabulary of our trained skip-gram model.

1.5.3 Conversion from KEGG ID to MeSH ID

In AsuratDB and the KEGG API, which is used to obtain drug-gene relations, genes are represented by gene IDs, while drugs are represented by KEGG IDs. Therefore, to use the data in BioConceptVec, we need to convert the KEGG IDs to MeSH IDs. We followed a four-step procedure (I), (II), (III), and (IV) to convert KEGG IDs to MeSH IDs.

(I) KEGG ID to PubChem SID or ChEBI ID Some drugs in KEGG are manually linked to external databases such as PubChem (`https://pubchem.ncbi.nlm.nih.gov/`) and ChEBI (`https://www.ebi.ac.uk/chebi/`), which are larger databases than KEGG. Therefore, we used these databases to link their IDs to MeSH IDs.

Since PubChem is maintained by the NCBI (National Center for Biotechnology Information, `https://www.ncbi.nlm.nih.gov/`) that also maintains the MeSH database, we prioritized the conversion of KEGG IDs to PubChem Substance IDs

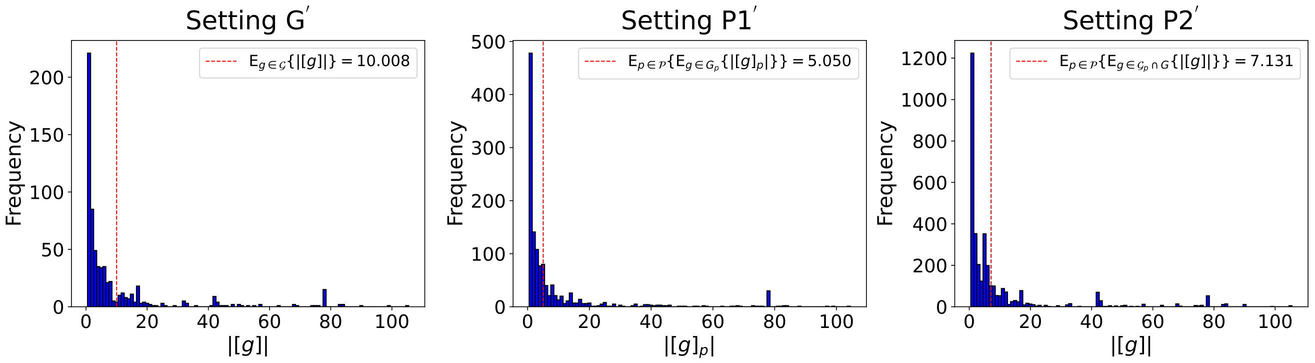

(a) BioConceptVec

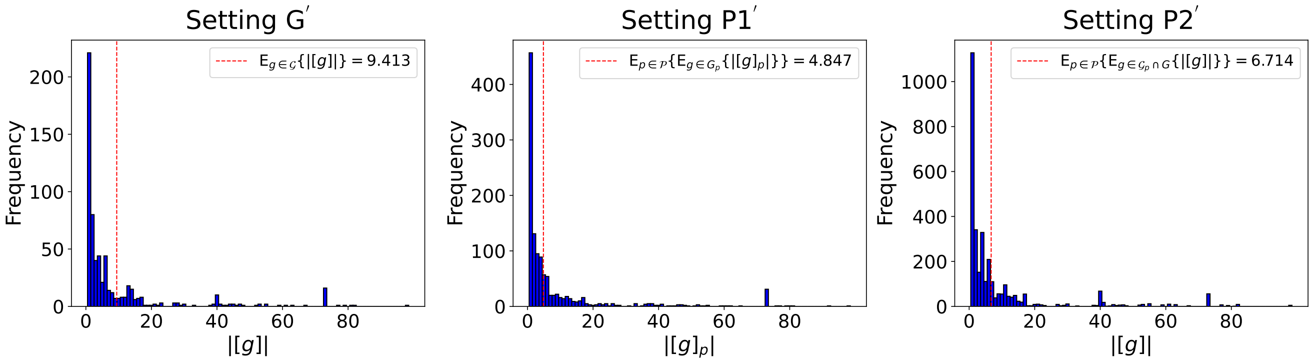

(b) Our embeddings

Figure S3. Distribution of the sizes of the answer sets for each gene g in the settings for predicting drugs from genes. The mean values are also shown: for setting G′, the value of $E_{g\in G}\{|[g]|\}$ from Table 2; for setting P1′, the value of $E_{p\in \mathscr{P}}\{E_{g\in G_p}\{|[g]_p|\}\}$ from Table S1; and for setting P2′, the value of $E_{p\in \mathscr{P}}\{E_{g\in \mathscr{G}_p\cap G}\{|[g]|\}\}$ from Table S1.

Model	Version
GPT-3.5	gpt-3.5-turbo-0125
GPT-4	gpt-4-turbo-2024-04-09
GPT-4o	gpt-4o-2024-05-13

Table S5. Version of each GPT model.

(SIDs). If we could not convert KEGG IDs directly to PubChem SIDs, we converted them to ChEBI IDs. The conversion from KEGG IDs to PubChem SIDs is available at https://rest.kegg.jp/conv/pubchem/drug, and the conversion to ChEBI IDs is available at https://rest.kegg.jp/conv/chebi/drug.

(II) PubChem SID or ChEBI ID to PubChem CID Next, we converted PubChem SIDs and ChEBI IDs to PubChem Compound IDs (CIDs), which are associated with MeSH IDs. The conversion from PubChem SIDs to PubChem CIDs is available at https://pubchem.ncbi.nlm.nih.gov/rest/pug/substance/sourceall/KEGG/cids/json. To convert ChEBI IDs to PubChem CIDs, we used the PubChem API. For example, for ChEBI ID 39112, the corresponding data can be obtained at https://pubchem.ncbi.nlm.nih.gov/rest/pug//compound/xref/RegistryID/chebi:39112/cids/json.

(III) Convert CIDs to MeSH Headings We used the Entrez (https://www.ncbi.nlm.nih.gov/Web/Search/entrezfs.html) System API provided by NCBI, and obtained the MeSH headings associated with each CID. This API allows for batch requests. For example, for CIDs 5328940 and 156413, the corresponding data can be obtained from the following URL: https://eutils.ncbi.nlm.nih.gov/entrez/eutils/esummary.fcgi?db=pccompound&id=5328940,156413&retmode=json.

Role	Prompt
system	You are a biologist specializing in drug-target interactions. Your responses should be formatted as JSON, with data listed under the key ‘target_genes’.
user	List the top 10 potential target genes for the drug *drug name*.

Table S6. Prompt template used for the GPT series to predict the top 10 target genes for each query drug. Here, *drug name* is a specific name such as Bosutinib.

Model	Setting	Method	Centering	Top1	Top10	MRR
Our embeddings	G	Random		0.020	0.140	0.065
		$\hat{\mathbf{v}}_{\text{naive}}$		0.067	0.197	0.111
			✓	0.125	0.381	0.209
		$\hat{\mathbf{v}}$		0.206	0.455	0.289
			✓	**0.300**	**0.686**	**0.426**
	P1	Random		0.020	0.142	0.066
		$\hat{\mathbf{v}}_{p,\text{naive}}$		0.364	0.661	0.470
			✓	0.455	0.810	0.581
		$\hat{\mathbf{v}}_p$		0.521	0.788	0.615
			✓	**0.589**	**0.862**	**0.685**
	P2	Random		0.023	0.164	0.074
		$\hat{\mathbf{v}}_{p,\text{naive}}$		0.376	0.682	0.484
			✓	0.473	0.837	0.601
		$\hat{\mathbf{v}}_p$		0.530	0.803	0.626
			✓	**0.600**	**0.880**	**0.700**

Table S7. Results of the naive estimators and the centering ablation study for settings G, P1, and P2.

(IV) MeSH Headings to MeSH ID Since we had already obtained the correspondence between MeSH IDs and MeSH headings in Supplementary Information 1.5.1, we could match MeSH IDs with the MeSH headings obtained through the Entrez system API. In this way, we converted the original KEGG IDs to MeSH IDs. Note that multiple KEGG IDs may correspond to the same MeSH ID. In such cases, we chose the smallest KEGG ID to associate with the MeSH ID.

1.6 Details of predictions using random baseline

This section describes the sets used for sampling in the random baseline across different settings. For settings G, P1, and P2, the set used was G. Similarly, D was used for G$'$, P1$'$, and P2$'$, while G^y was used for settings Y1, P1Y1, and P2Y1. For settings Y2, P1Y2, and P2Y2, $G^{y|L_y} \cup G^{y|U_y}$ was used instead of $G^{y|U_y}$; This was intended to prevent extremely high random baseline performance when the size of $G^{y|U_y}$ is very small.

1.7 Details of predictions using the GPT series

In this study, we used GPT-3.5, GPT-4, and GPT-4o as baselines for generative models. The details of the models are shown in Table S5. The prompts are shown in Table S6.

1.8 Details of predictions by TransE

This section describes the details of the experimental settings for TransE. Embeddings are learned for each of settings G, P1, and P2. In our experiments, we utilized TransE with the following hyperparameters: a batch size of 1024, embedding dimensions set to 500, a learning rate of 0.0001, and a total of 800 iteration steps. Additionally, we adopted the self-adversarial negative sampling method from RotatE[2], configuring the negative sampling size to 512 and the sampling temperature to 0.5. For each setting, the triplets of drug d, gene g, and drug-gene relation (d,g) are used to evaluate performance in analogy tasks and are then split into 60% training, 20% validation, and 20% test data. In setting G, only a single type of relation, the drug-gene relation, is considered. In settings P1 and P2, the drug-gene relation (d,g) is distinguished based on each pathway $p \in \mathscr{P}$, resulting in $|\mathscr{P}|$ different relations. For entities in the validation and test data that do not appear in the training data, random embeddings are simply used. Since KGE typically uses the entire set of head and tail entities as its search space, we similarly define the search space for TransE as the entire set of drugs and genes that have drug-gene relations for each setting. Additionally, note that in Table 3, while random baseline, GPT series, and our method evaluate performance on the entire dataset, TransE evaluates performance on the 20% test data.

Model	Setting	Method	Metric		
			Top1	Top10	MRR
BioConceptVec	G'	Random	0.011	0.075	0.036
		$\hat{\mathbf{v}}'$	0.233	0.560	0.345
	$P1'$	Random	0.010	0.065	0.032
		$\hat{\mathbf{v}}'_p$	0.426	0.679	0.515
	$P2'$	Random	0.009	0.066	0.031
		$\hat{\mathbf{v}}'_p$	0.248	0.507	0.337
Our embeddings	G'	Random	0.010	0.078	0.037
		$\hat{\mathbf{v}}'$	0.240	0.577	0.351
	$P1'$	Random	0.012	0.070	0.034
		$\hat{\mathbf{v}}'_p$	0.478	0.751	0.571
	$P2'$	Random	0.009	0.070	0.033
		$\hat{\mathbf{v}}'_p$	0.279	0.516	0.359

Table S8. Gene prediction performance in settings G′, P1′, and P2′.

2 Details of experimental results

2.1 Analogy tasks for drug-gene pairs

2.1.1 Comparison of estimators and centering ablation study

For settings G, P1, and P2, Table S7 shows the results of the naive estimators defined in equations (5) and (S3) and the centering ablation study, using our skip-gram. In all settings, the results of the estimators $\hat{\mathbf{v}}$ and $\hat{\mathbf{v}}_p$ calculated from drug-gene relations outperformed those of the naive estimators $\hat{\mathbf{v}}_{\text{naive}}$ and $\hat{\mathbf{v}}_{p,\text{naive}}$. We also observed performance improvements due to centering. Based on this, we showed the results using the estimators calculated from the drug-gene relations with centering applied in Table 3.

2.1.2 Prediction of drugs from genes

As seen in Supplementary Information 1.3, Table S3 defines settings G', $P1'$, and $P2'$ for the analogy tasks for predicting drugs from genes. We then define estimators to perform the analogy tasks in these settings. Similar to the estimator $\hat{\mathbf{v}}$ in Eq. (7) for setting G, we define the estimator $\hat{\mathbf{v}}'$ for setting G':

$$\hat{\mathbf{v}}' := -\hat{\mathbf{v}} = \mathrm{E}_{\mathscr{R}}\{\mathbf{u}_d - \mathbf{u}_g\} = \frac{1}{|\mathscr{R}|} \sum_{(d,g)\in\mathscr{R}} (\mathbf{u}_d - \mathbf{u}_g). \tag{S25}$$

Similar to the estimator $\hat{\mathbf{v}}_p$ in Eq. (14) for settings P1 and P2, we also define the estimator $\hat{\mathbf{v}}'_p$ for settings $P1'$ and $P2'$:

$$\hat{\mathbf{v}}'_p := -\hat{\mathbf{v}}_p = \mathrm{E}_{\mathscr{R}_p}\{\mathbf{u}_d - \mathbf{u}_g\} = \frac{1}{|\mathscr{R}_p|} \sum_{(d,g)\in\mathscr{R}_p} (\mathbf{u}_d - \mathbf{u}_g). \tag{S26}$$

For settings G', $P1'$, and $P2'$, we performed the analogy tasks using $\hat{\mathbf{v}}'$ and $\hat{\mathbf{v}}'_p$, and Table S8 shows the results. Similar to Table 3, the estimators calculated from the drug-gene relations outperformed the random baseline.

While Table 3 shows higher scores for setting P2 compared to setting P1, Table S8 shows higher scores for setting P1′ compared to setting P2′. To explain these results, we focus on the sizes of the query and answer sets, using actual values from BioConceptVec. As shown in Table S1, the sizes of the query sets for settings P1 and P2 are $\sum_{p\in\mathscr{P}} |D_p| = 4087$ and $\sum_{p\in\mathscr{P}} |\mathscr{D}_p \cap D| = 4091$, respectively. The ratio is $\sum_{p\in\mathscr{P}} |\mathscr{D}_p \cap D| / \sum_{p\in\mathscr{P}} |D_p| \approx 1.001$. On the other hand, for settings $P1'$ and $P2'$, the sizes of the query sets are $\sum_{p\in\mathscr{P}} |G_p| = 1251$ and $\sum_{p\in\mathscr{P}} |\mathscr{G}_p \cap G| = 3463$, respectively, with a ratio of $\sum_{p\in\mathscr{P}} |\mathscr{G}_p \cap G| / \sum_{p\in\mathscr{P}} |G_p| \approx 2.768$. For settings P1, P2, P1′, and P2′, the answer sets defined in Tables S2 and S3 are $[d]_p$, $[d]$, and $[g]_p$, $[g]$, respectively. By definition, the sizes of the answer sets satisfy $|[d]_p| \leq |[d]|$ and $|[g]_p| \leq |[g]|$. In fact, the expected values of them are $\mathrm{E}_{p\in\mathscr{P}}\{\mathrm{E}_{d\in D_p}\{|[d]_p|\}\} = 1.965$, $\mathrm{E}_{d\in D}\{|[d]|\} = 2.938$, $\mathrm{E}_{p\in\mathscr{P}}\{\mathrm{E}_{g\in G_p}\{|[g]_p|\}\} = 5.050$, and $\mathrm{E}_{g\in G}\{|[g]|\} = 10.008$, respectively. In settings P1 and P2, the sizes of the query sets are nearly identical, but the larger answer sets in setting P2 probably make the task easier. On the other hand, the sizes of the query sets in setting $P2'$ are more than twice as large as those in $P1'$, which likely contributes to the lower evaluation metric scores in setting $P2'$.

Vocabulary	Setting	LLM	Metric	
			Top1	Top10
BioConceptVec	G	GPT-3.5	0.611	0.785
		GPT-4	0.665	0.795
		GPT-4o	0.718	0.848
	P1	GPT-3.5	0.581	0.780
		GPT-4	0.633	0.792
		GPT-4o	0.689	0.855
	P2	GPT-3.5	0.627	0.803
		GPT-4	0.685	0.813
		GPT-4o	0.742	0.873
Our embeddings	G	GPT-3.5	0.637	0.804
		GPT-4	0.693	0.818
		GPT-4o	0.741	0.867
	P1	GPT-3.5	0.604	0.796
		GPT-4	0.654	0.808
		GPT-4o	0.703	0.868
	P2	GPT-3.5	0.653	0.821
		GPT-4	0.710	0.832
		GPT-4o	0.760	0.887

Table S9. Gene prediction performance for GPT models.

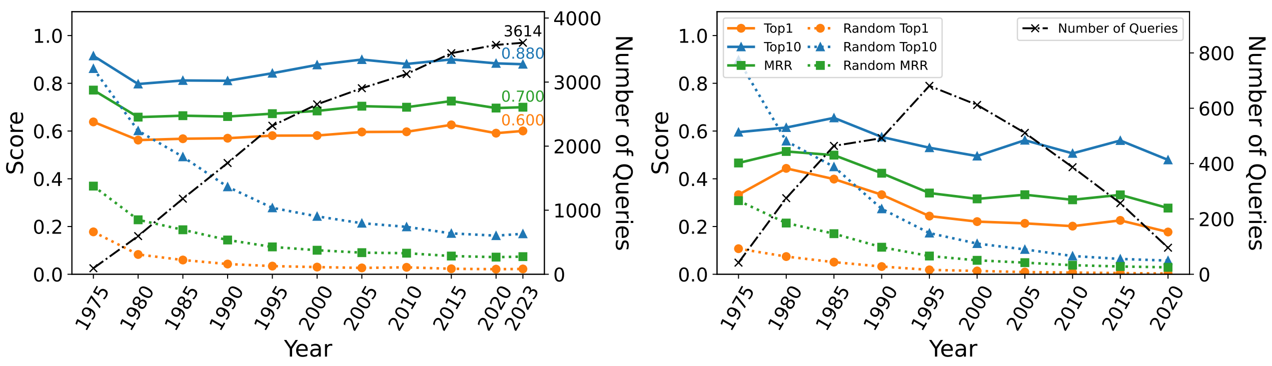

(a) P2Y1

(b) P2Y2

Figure S4. Gene prediction performance and the number of queries for each year in settings P2Y1 and P2Y2.

2.1.3 Predictions by GPT models

The prediction results from the GPT models are shown in Table S9. The set of query drugs and the set of correct target genes depend not only on settings G, P1, and P2 but also on the vocabulary set. Here, we conducted experiments using vocabularies obtained from BioConceptVec or our skip-gram embeddings. The best accuracies for all combinations of vocabularies and settings are summarized in the Table 3 in the main text.

2.2 Analogy tasks for drug-gene pairs by year

2.2.1 Global setting by year

Tables S10 and S11 show the statistics for settings Y1 and Y2. As seen in Table S2, in setting Y1, the query is $d \in D^y$, and the search space is $\mathscr{G}^y$. In setting Y2, the query is $d \in D^{y|U_y}$, and the search space is $\mathscr{G}^y \setminus [d]^{y|L_y}$. In Table S10, D^y and $\mathscr{G}^y$ show a monotonic increase over the years. On the other hand, Table S11 shows that $D^{y|U_y}$ has increased since 1975 and started to decrease after 1995.

We showed the plots of the results for settings Y1 and Y2 in Fig. 3. For settings Y1 and Y2, Table S12 shows detailed

	Year										
	1975	1980	1985	1990	1995	2000	2005	2010	2015	2020	2023
$\lvert\mathscr{D}^y\rvert$	3275	5059	7125	9387	11748	14044	16827	19758	22717	25431	28284
$\lvert\mathscr{G}^y\rvert$	725	1782	3413	5976	10533	16949	24784	32264	39961	47509	51057
$\lvert\mathscr{R}^y\rvert$	128	429	807	1251	2337	3282	4054	4849	5551	5909	5968
$\lvert D^y\rvert$	104	343	666	904	1253	1436	1566	1708	1875	1961	1980
$\lvert G^y\rvert$	21	47	75	125	228	348	430	521	590	630	634
$\mathrm{E}_{d\in D^y}\{\lvert[d]\rvert\}$	1.231	1.251	1.212	1.384	1.865	2.286	2.589	2.839	2.961	3.013	3.014
$\mathrm{E}_{g\in G^y}\{\lvert[g]\rvert\}$	6.095	9.128	10.760	10.008	10.250	9.431	9.428	9.307	9.408	9.379	9.413

Table S10. Statistics for setting Y1.

	Year									
	1975	1980	1985	1990	1995	2000	2005	2010	2015	2020
$\lvert\mathscr{R}^{y\mid L_y}\rvert$	37	117	256	499	859	1269	1637	1978	2354	2603
$\lvert\mathscr{R}^{y\mid U_y}\rvert$	53	183	296	344	515	473	379	281	189	61
$\lvert D^{y\mid U_y}\rvert$	48	154	272	288	369	325	256	189	131	48
$\lvert G^{y\mid U_y}\rvert$	14	34	55	89	137	173	153	140	115	44
$\mathrm{E}_{d\in D^{y\mid L_y}}\left\{\left\lvert[d]^{y\mid L_y}\right\rvert\right\}$	1.028	1.104	1.133	1.188	1.372	1.500	1.613	1.665	1.722	1.760
$\mathrm{E}_{d\in D^{y\mid U_y}}\left\{\left\lvert[d]^{y\mid U_y}\right\rvert\right\}$	1.104	1.188	1.088	1.194	1.396	1.455	1.480	1.487	1.443	1.271
$\mathrm{E}_{g\in G^{y\mid L_y}}\left\{\left\lvert[g]^{y\mid L_y}\right\rvert\right\}$	2.467	4.179	5.020	5.940	5.335	4.789	4.547	4.425	4.467	4.591
$\mathrm{E}_{g\in G^{y\mid U_y}}\left\{\left\lvert[g]^{y\mid U_y}\right\rvert\right\}$	3.786	5.382	5.382	3.865	3.759	2.734	2.477	2.007	1.643	1.386

Table S11. Statistics for setting Y2.

results used in Fig. 3.

2.2.2 Pahtway-wise setting by year

Tables S13 and S14 show the statistics for settings P1Y1, P2Y1, P1Y2, and P2Y2. As seen in Table S2, the query is $d \in D_p^y$ in setting P1Y1, $d \in \mathscr{D}_p^y \cap D^y$ in setting P2Y1, $d \in D_p^{y|U_y}$ in setting P1Y2, and $d \in \mathscr{D}_p^y \cap D^{y|U_y}$ in P2Y2. In Table S13, D_p^y and $\mathscr{D}_p^y \cap D^y$ show a monotonic increase over the years. On the other hand, Table S14 shows that $D_p^{y|U_y}$ and $\mathscr{D}_p^y \cap D^{y|U_y}$ have increased since 1975 and started to decrease after 1995.

We showed the plots of the results for settings P1Y1 and P1Y2 in Fig. 3. Fig. S4 shows the plots of the results for settings P2Y1 and P2Y2. For settings P1Y1, P2Y1, P1Y2, and P2Y2, Table S15 shows detailed results used in Fig. 3 and Fig. S4. As shown in Table 3, settings P1 and P2 show roughly the same trends. Similarly, in Table S15, settings P1Y1 and P2Y1, as well as P1Y2 and P2Y2, each show similar trends.

2.3 Correlation between answer set sizes and predicted ranks

Fig. S5 shows scatter plots and correlation coefficients between the answer set sizes for each drug d and the ranks of the predicted genes obtained by adding relation vectors in settings G, P1, and P2. Since smaller answer set sizes are more frequent (see Fig. S2 in Supplementary Information 1.4 for the distribution of answer set sizes), we calculated a weighted Pearson correlation coefficient, where the weight is the reciprocal of the frequency of each answer set size.

2.4 Details of comparison of analogy computation and TransE

This section provides details regarding the experiments presented in Fig. 4, comparing our analogy computation with TransE. We conducted experiments under setting P1, which is a simple setting and involves multiple drug-gene relations. For drug-gene relations in Setting P1, we fixed 20% of the data as validation data and another 20% as test data, following the same setting for TransE in Table 3, and used them consistently across both methods. From the remaining 60% of the data, we created training subsets ranging from 10% to 60%, ensuring that each smaller subset is contained within the next larger subset. Formally, if we define the 10% to 60% training sets as $\mathscr{T}_{10},\ldots,\mathscr{T}_{60}$, then $\mathscr{T}_{10} \subset \mathscr{T}_{20} \subset \mathscr{T}_{30} \subset \mathscr{T}_{40} \subset \mathscr{T}_{50} \subset \mathscr{T}_{60}$.

Metric	Setting	Method	Year 1975	1980	1985	1990	1995	2000	2005	2010	2015	2020	2023
Top1	Y1	Random	0.143	0.066	0.054	0.036	0.027	0.022	0.024	0.022	0.022	0.019	0.019
		$\hat{\mathbf{v}}^y$	0.365	0.262	0.249	0.236	0.247	0.272	0.275	0.287	0.323	0.301	0.300
	Y2	Random	0.104	0.081	0.043	0.028	0.016	0.012	0.013	0.006	0.009	0.004	–
		$\hat{\mathbf{v}}^{y\|L_y}$	0.333	0.208	0.165	0.132	0.076	0.092	0.062	0.090	0.107	0.021	–
Top10	Y1	Random	0.830	0.554	0.426	0.317	0.236	0.196	0.185	0.165	0.153	0.137	0.140
		$\hat{\mathbf{v}}^y$	0.837	0.577	0.563	0.562	0.578	0.652	0.691	0.651	0.714	0.693	0.686
	Y2	Random	0.835	0.538	0.382	0.253	0.157	0.123	0.098	0.064	0.062	0.081	–
		$\hat{\mathbf{v}}^{y\|L_y}$	0.625	0.487	0.438	0.410	0.347	0.397	0.352	0.344	0.328	0.271	–
MRR	Y1	Random	0.340	0.204	0.167	0.127	0.100	0.084	0.081	0.073	0.069	0.064	0.064
		$\hat{\mathbf{v}}^y$	0.534	0.370	0.353	0.346	0.363	0.399	0.410	0.410	0.455	0.430	0.426
	Y2	Random	0.305	0.211	0.150	0.104	0.070	0.055	0.049	0.034	0.034	0.032	–
		$\hat{\mathbf{v}}^{y\|L_y}$	0.438	0.304	0.253	0.214	0.163	0.182	0.147	0.173	0.188	0.103	–

Table S12. Gene prediction performance in settings Y1 and Y2.

	Year 1975	1980	1985	1990	1995	2000	2005	2010	2015	2020	2023
$\sum_{p\in\mathscr{P}} \|D_p^y\|$	85	536	1092	1668	2271	2609	2897	3122	3447	3578	3612
$\sum_{p\in\mathscr{P}} \|G_p^y\|$	15	76	127	234	444	634	765	916	1088	1167	1178
$\sum_{p\in\mathscr{P}} \|\mathscr{D}_p^y \cap D^y\|$	397	1015	1559	2055	2394	2690	2909	3132	3458	3581	3614
$\sum_{p\in\mathscr{P}} \|\mathscr{G}_p^y \cap G^y\|$	25	136	271	553	1242	1896	2325	2570	2952	3174	3186
$\mathrm{E}_{p\in\mathscr{P}}\{\mathrm{E}_{d\in D_p^y}\{\|[d]_p^y\|\}\}$	1.078	1.446	1.289	1.341	1.603	1.803	1.841	2.027	1.983	1.979	1.990
$\mathrm{E}_{p\in\mathscr{P}}\{\mathrm{E}_{g\in G_p^y}\{\|[g]_p^y\|\}\}$	6.143	8.485	8.084	7.485	7.027	6.032	5.607	5.529	4.973	4.830	4.847
$\mathrm{E}_{p\in\mathscr{P}}\{\mathrm{E}_{d\in\mathscr{D}_p^y\cap D^y}\{\|[d]^y\|\}\}$	1.078	1.430	1.356	1.616	2.211	2.532	2.724	2.902	2.749	2.759	2.776
$\mathrm{E}_{p\in\mathscr{P}}\{\mathrm{E}_{g\in\mathscr{G}_p^y\cap G^y}\{\|[g]^y\|\}\}$	10.000	13.496	12.787	11.633	8.815	7.163	6.968	6.505	6.525	6.692	6.714

Table S13. Statistics for settings P1Y1 and P2Y1.

We explain the experimental settings of our method. We used our skip-gram embeddings. At test time, for pathways present in the training data, we used the relation vector $\hat{\mathbf{v}}_p$ defined in Eq. (14). For pathways not present in the training data, we used the relation vector $\hat{\mathbf{v}}$ computed from all drug-gene relations in the training data, as defined in Eq. (7). The search space consisted of all genes appearing in the training, validation, and test data.

The experimental settings for TransE follow those described in Supplementary Information 1.8.

References

1. Maglott, D., Ostell, J., Pruitt, K. D. & Tatusova, T. Entrez gene: gene-centered information at ncbi. *Nucleic acids research* **33**, D54–D58 (2005).
2. Sun, Z., Deng, Z.-H., Nie, J.-Y. & Tang, J. Rotate: Knowledge graph embedding by relational rotation in complex space. *arXiv preprint arXiv:1902.10197* (2019).

	Year									
	1975	1980	1985	1990	1995	2000	2005	2010	2015	2020
$\sum_{p\in\mathscr{P}} \left\lvert D_p^{y\lvert U_y} \right\rvert$	40	253	419	448	565	500	426	303	195	66
$\sum_{p\in\mathscr{P}} \left\lvert G_p^{y\lvert U_y} \right\rvert$	10	58	90	154	272	295	250	218	163	61
$\sum_{p\in\mathscr{P}} \lvert \mathscr{D}_p^y \cap D^{y\lvert U_y} \rvert$	42	275	464	492	681	612	511	387	257	96
$\sum_{p\in\mathscr{P}} \lvert \mathscr{G}_p^y \cap G^{y\lvert U_y} \rvert$	14	68	121	250	531	607	589	549	496	206
$\mathrm{E}_{p\in\mathscr{P}} \left\{ \mathrm{E}_{d\in D_p^{y\lvert U_y}} \left\{ \left\lvert [d]_p^{y\lvert U_y} \right\rvert \right\} \right\}$	1.034	1.445	1.247	1.285	1.477	1.571	1.596	1.724	1.648	1.614
$\mathrm{E}_{p\in\mathscr{P}} \left\{ \mathrm{E}_{g\in G_p^{y\lvert U_y}} \left\{ \left\lvert [g]_p^{y\lvert U_y} \right\rvert \right\} \right\}$	4.194	7.157	6.560	5.761	5.107	4.329	3.924	3.884	3.538	3.443
$\mathrm{E}_{p\in\mathscr{P}} \left\{ \mathrm{E}_{d\in \mathscr{D}_p^y \cap D^{y\lvert U_y}} \left\{ \left\lvert [d]^{y\lvert U_y} \right\rvert \right\} \right\}$	1.034	1.427	1.301	1.497	1.925	2.126	2.288	2.376	2.176	2.113
$\mathrm{E}_{p\in\mathscr{P}} \left\{ \mathrm{E}_{g\in \mathscr{G}_p^y \cap G^{y\lvert U_y}} \left\{ \left\lvert [g]^{y\lvert U_y} \right\rvert \right\} \right\}$	7.000	10.525	9.027	7.890	5.667	4.729	4.392	4.155	4.096	4.249

Table S14. Statistics for settings P1Y2 and P2Y2.

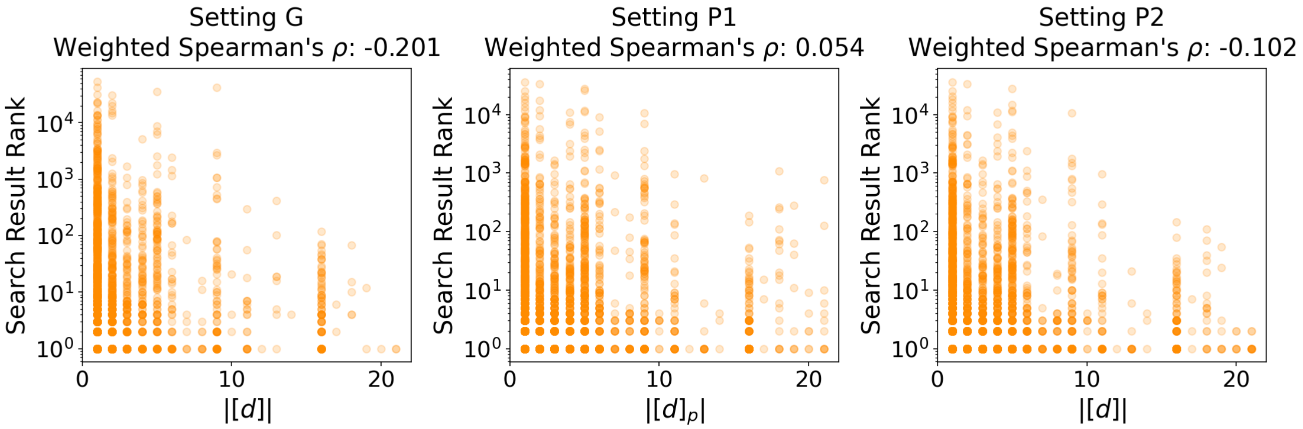

(a) BioConceptVec

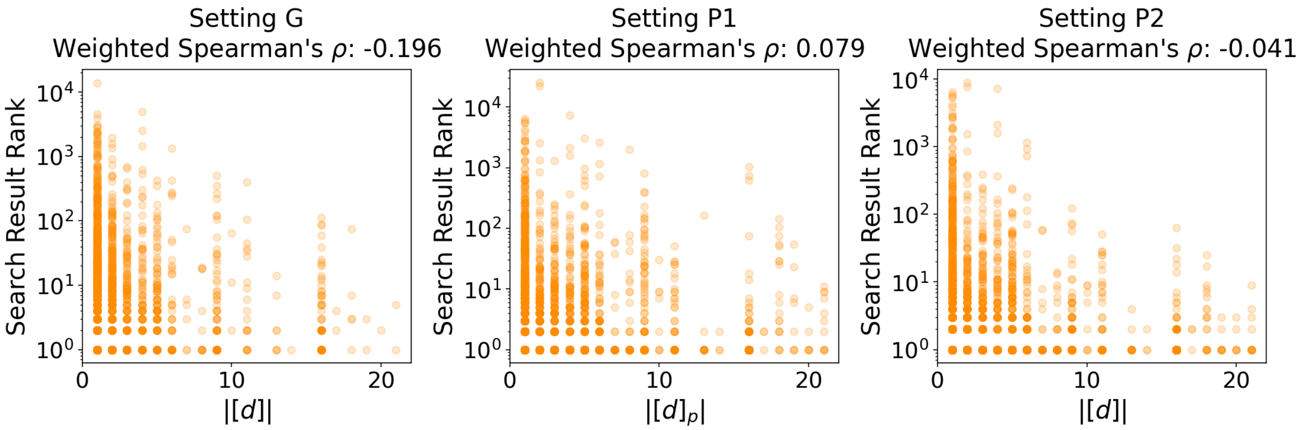

(b) Our embeddings

Figure S5. Scatter plots of the answer set sizes for each drug d and the ranks of the predicted genes.

Metric	Setting	Method	1975	1980	1985	1990	1995	Year<br>2000	2005	2010	2015	2020	2023
Top1	P1Y1	Random	0.151	0.086	0.062	0.044	0.028	0.025	0.022	0.021	0.021	0.019	0.020
		$\hat{\mathbf{v}}_p^y$	0.706	0.621	0.613	0.592	0.589	0.588	0.588	0.587	0.614	0.582	0.589
	P2Y1	Random	0.178	0.083	0.060	0.043	0.034	0.030	0.027	0.029	0.023	0.021	0.022
		$\hat{\mathbf{v}}_p^y$	0.638	0.562	0.568	0.570	0.581	0.581	0.596	0.597	0.626	0.591	0.600
	P1Y2	Random	0.147	0.079	0.054	0.033	0.017	0.012	0.013	0.007	0.004	0.006	–
		$\hat{\mathbf{v}}_p^{y\|L_y}$	0.350	0.482	0.442	0.366	0.290	0.266	0.254	0.244	0.272	0.258	–
	P2Y2	Random	0.107	0.074	0.051	0.032	0.019	0.014	0.009	0.007	0.005	0.003	–
		$\hat{\mathbf{v}}_p^{y\|L_y}$	0.333	0.444	0.399	0.333	0.244	0.221	0.213	0.202	0.226	0.177	–
Top10	P1Y1	Random	0.906	0.602	0.486	0.351	0.245	0.214	0.186	0.165	0.146	0.140	0.142
		$\hat{\mathbf{v}}_p^y$	1.000	0.871	0.862	0.835	0.847	0.881	0.889	0.867	0.886	0.869	0.862
	P2Y1	Random	0.862	0.601	0.492	0.366	0.278	0.243	0.214	0.199	0.171	0.162	0.170
		$\hat{\mathbf{v}}_p^y$	0.915	0.797	0.812	0.811	0.842	0.877	0.899	0.881	0.900	0.884	0.880
	P1Y2	Random	0.922	0.583	0.448	0.269	0.156	0.118	0.098	0.068	0.061	0.076	–
		$\hat{\mathbf{v}}_p^{y\|L_y}$	0.625	0.648	0.704	0.621	0.586	0.564	0.620	0.558	0.662	0.606	–
	P2Y2	Random	0.898	0.558	0.450	0.274	0.173	0.129	0.104	0.076	0.064	0.057	–
		$\hat{\mathbf{v}}_p^{y\|L_y}$	0.595	0.615	0.655	0.575	0.530	0.495	0.562	0.506	0.560	0.479	–
MRR	P1Y1	Random	0.366	0.231	0.186	0.140	0.102	0.090	0.080	0.073	0.068	0.064	0.065
		$\hat{\mathbf{v}}_p^y$	0.849	0.724	0.712	0.684	0.680	0.690	0.694	0.688	0.713	0.684	0.685
	P2Y1	Random	0.369	0.228	0.186	0.144	0.115	0.101	0.091	0.088	0.076	0.072	0.074
		$\hat{\mathbf{v}}_p^y$	0.772	0.658	0.664	0.661	0.673	0.684	0.704	0.700	0.726	0.696	0.700
	P1Y2	Random	0.358	0.224	0.169	0.113	0.071	0.054	0.049	0.034	0.031	0.034	–
		$\hat{\mathbf{v}}_p^{y\|L_y}$	0.488	0.555	0.547	0.459	0.392	0.370	0.384	0.362	0.400	0.382	–
	P2Y2	Random	0.308	0.215	0.170	0.113	0.076	0.058	0.049	0.038	0.033	0.029	–
		$\hat{\mathbf{v}}_p^{y\|L_y}$	0.466	0.514	0.499	0.423	0.340	0.316	0.333	0.312	0.333	0.277	–

Table S15. Gene prediction performance in settings P1Y1, P2Y1, P1Y2, and P2Y2.
